# LCZ696 Attenuated Doxorubicin-Induced Chronic Cardiomyopathy Through the TLR2-MyD88 Complex Formation

**DOI:** 10.3389/fcell.2021.654051

**Published:** 2021-04-13

**Authors:** Shiju Ye, Lan Su, Peiren Shan, Bozhi Ye, Shengjie Wu, Guang Liang, Weijian Huang

**Affiliations:** ^1^Department of Cardiology, The First Affiliated Hospital of Wenzhou Medical University, Wenzhou, China; ^2^The Key Laboratory of Cardiovascular Disease of Wenzhou, Wenzhou, China; ^3^Chemical Biology Research Center, School of Pharmaceutical Sciences, Wenzhou Medical University, Wenzhou, China

**Keywords:** doxorubicin, LZC696, toll-like receptors, heart failure, molecular target

## Abstract

**Background and Purpose:**

The profibrotic and proinflammatory effects induced by doxorubicin (DOX) are key processes in the development of serious heart damage. Lack of effective drugs and the unclear mechanisms of its side effects limit the clinical treatment of DOX-induced cardiac injury. This study aimed to explore the protective role of LCZ696 and the potential mechanism of Toll-like receptor 2 (TLR2) in doxorubicin-induced cardiac failure.

**Experimental Approach:**

DOX (5 mg/kg/week, three times) was used to establish a chronic cardiomyopathy mouse model. Heart function tests, pathology examinations and molecular biology analyses were used to explore the effects of LCZ696 and TLR2 deficiency *in vivo* and *in vitro*. Computational docking was applied to predict the key residues for protein-ligand interaction.

**Key Results:**

The EF% declined, and the LVIDd, pro-fibrosis marker levels and NF-κB related inflammatory response increased in the chronic cardiomyopathy group induced by DOX. LCZ696 treatment and TLR2 deficiency reversed these heart damage *in vivo*. In H9C2 cells, pre-treatment with LCZ696 and TLR2 knockdown suppressed the DOX-induced high expression of profibrotic and proinflammatory markers. Moreover, DOX notably increased the TLR2-MyD88 interaction *in vivo* and *in vitro*, which was inhibited by LCZ696. Finally, we demonstrated the direct interaction between DOX and TLR2 via hydrogen bonds on Pro-681 and Glu-727 and Pro-681 and Ser-704 may be the key residues by which LCZ696 affects the interaction between DOX and TLR2.

**Conclusion and Implications:**

LCZ696 prevents DOX-induced cardiac dilation failure, fibrosis and inflammation by reducing the formation of TLR2-MyD88 complexes. LZC696 may be a potential effective drug to treat DOX-induced heart failure.

## Introduction

Doxorubicin (DOX), a member of the family of anthracyclines and an antitumor antibiotic, has been widely used to treat breast cancer, bladder cancer, and so on (Tacar et al., 2013). However, it has been well documented that the most dangerous side effect of doxorubicin is cardiovascular toxicity, and the clinical manifestations are as follows, such as tachycardia, hypotension, arrhythmias, and dilated cardiomyopathy (DCM), which leads to congestive heart failure (HF) (Chatterjee et al., 2010; Wallace et al., 2020). Notably, this side effect is positively correlated with the cumulative dose of DOX. As previously reported, the incidence of DOX cardiomyopathy increases to 36% when the dose exceeds 600 mg/m^2^ (Lefrak et al., 1973), and the incidence of congestive HF affects 26% of the patients receiving DOX when the cumulative dose exceeds 550 mg/m^2^ (Swain et al., 2003).

Previous studies have suggested that doxorubicin causes cardiomyopathy related to oxidative stress, downregulation of contractile protein genes and p53-mediated apoptosis (Vejpongsa and Yeh, 2014). Recent studies have found that doxorubicin has a strong inflammatory effect, which mainly manifests as doxorubicin further promoting the expression of the following cardiac inflammatory factors: (1) DOX indirectly induces interleukin 6 (IL-6) and tumor necrosis factor-α (TNF-α) through autocrine and paracrine processes; (2) DOX promotes cardiac fibroblast proliferation and extracellular matrix protein synthesis; and (3) Toll-like receptor-4, PI3Kγ and other inflammatory mediators are activated, which leads to a vicious cycle of inflammatory reactions in cardiac cells (Riad et al., 2008; Li et al., 2018). Increasing attention should be paid to the inflammatory response to cardiomyopathy caused by doxorubicin.

LCZ696, also known as the sacubitril/valsartan, consists of the neprilysin inhibitor sacubitril and the angiotensin receptor blocker valsartan, is a combination drug for use in patients with HF and a reduced ejection fraction (Lillyblad, 2015; Hubers and Brown, 2016; Yancy et al., 2016). Recently, many studies have explored the anti-inflammatory effect of LCZ696 in basic research, with evidence that this drug can attenuate cardiac dysfunction after myocardial infarction (von Lueder et al., 2015), inhibit oxidative stress, inflammation, and fibrosis, improve renal function in CKD (Jing et al., 2017) and ameliorate NLRP3 after relieving the pressure overload in mice (Li X. et al., 2020). In addition, Xia et al. (2017) found that LCZ696 protects cardiac function from doxorubicin-induced DCM by alleviating Drp1-mediated mitochondrial dysfunction. However, the potential effect of LCZ696 on DOX-induced cardiac inflammation and cardiac dysfunction, especially the underlying mechanisms of its anti-inflammatory effects, remains to be elucidated.

Toll-like receptor 2 (TLR2) is one part of the family of Toll-like receptors (TLRs), which mainly mediate pathogen-induced inflammation in innate immunity (Henrick et al., 2016; Elshabrawy et al., 2017). Although there is no involvement of endotoxins such as viruses and bacteria, recent studies have demonstrated that TLR2 may play a potential role in the inflammatory response to the process of cardiac remodeling caused by DOX. Nozaki et al. (2004) found that TLR2 knockout mice exhibited preserved cardiac function and an increased survival rate compared to DOX-challenged mice through mediating cardiac inflammatory and apoptosis, and Liang et al. (2018) found that the levels of TLR2 were upregulated in doxorubicin-treated patients who developed heart dysfunction. Similarly, after analyzing a large amount of clinical data, Pop-Moldovan et al. (2017) found that the expression of TLR4 and TLR2 was higher in patients with diastolic dysfunction treated with doxorubicin. These findings indicated that TLR2 may play a role in the mediation of DOX-induced cardiomyopathy. However, how DOX activates TLR2-related inflammation and whether LCZ696 could attenuate DOX-induced cardiac failure in a TLR2-dependent manner remain unaddressed.

In this study, utilizing LCZ696 and TLR2 knockout mice, we investigated the effect of LCZ696 and TLR2 deficiency on DOX-induced mouse cardiomyopathy. Our results found that LCZ696 treatment and TLR2 deficiency reversed DOX-induced diastolic HF, cardiac fibrosis and inflammation. More interesting, we found that LCZ696 may directly inhibit the formation of the TLR2/MyD88 complex activated by DOX, which results in the attenuation of DOX-induced DCM.

## Materials and Methods

### Cell Culture and Reagents

Cultured H9C2 cell lines (immortalized rat cardiomyocytes) were obtained from the Shanghai Institute of Biochemistry and Cell Biology (Shanghai, China). The cells were cultured in Dulbecco’s modified Eagle 110 medium (DMEM) supplemented with 10% fetal bovine serum, 100 U/mL penicillin and 100 U/mL streptomycin at 37°C in a humidified 5% CO2 incubator.

Doxorubicin (D107159) was purchased from Aladdin (Los Angeles, CA, United States). LCZ696 (S7678) was obtained from Sellerk (Shanghai, China). Antibodies against IκBα (4812S), nuclear factor-κB (NF-κB) (p65) (8242S), and GADPH (5174) were purchased from Cell Signaling Technology (CST, United States), and antibodies against TLR2 (12276), TLR4 (ab22048), MyD88 (ab2064), COL-1 (ab34710), transforming growth factor (TGF-β) (ab92486), TNF-α (ab6671), and Lamin B (ab133741) were purchased from Abcam (Shanghai, China).

### DOX-Induced Chronic Cardiac Injury in Mice

All animal care and experimental procedures were approved by the Animal Policy and Welfare Committee of Wenzhou Medical University (Approval Document No. wydw2016-0124), and all animals received humane care according to the National Institutes of Health (United States) guidelines. All studies followed the ARRIVE guidelines for reporting experiments involving animals (Lilley et al., 2020; Percie du Sert et al., 2020).

C57BL/6 male mice were obtained from the Animal Center of Wenzhou Medical University, and male TLR2KO mice (B6.129-TLR2tm1Kir/J) backcrossed to C57BL/6 were provided by the Jackson Laboratory of America (Bar Harbor, ME, United States). The mice were housed with a 12:12 h light–dark cycle at a constant room temperature and fed a standard rodent diet. The mice were acclimatized to the laboratory for at least 2 weeks before initiating the studies. Detailed methods for the model are presented below. Sample sizes were defined by *a priori* power calculations with G-Power 3.1.9 software^1^, considering a statistical power of 80% and α = 0.05.

Eight-week-old C57BL/6 mice and TLR2KO mice were randomly divided into five groups: (I) untreated C57BL/6 mice receiving PBS (WT-Ctrl, *n* = 7); (II) DOX-injected C57BL/6 mice receiving PBS (WT-DOX; *n* = 7); (III) DOX-injected C57BL/6 mice treated orally with LCZ696 (60 mg/kg/day) (Suematsu et al., 2016) (WT-DOX + LCZ696; *n* = 7); (IV) uninjected TLR2 KO mice receiving PBS (TLR2KO-Ctrl; *n* = 7); and (V) DOX-injected TLR2 KO mice receiving PBS (TLR2KO-DOX; *n* = 7). DOX (5 mg/kg, once a week, total cumulative dose of 15 mg/kg) was administered three times via intraperitoneal injection as described previously (Pop-Moldovan et al., 2017). LCZ696 treatments were initiated 1 day after starting the DOX injections and continued throughout the 6-week follow-up. Six weeks after DOX treatment, cardiotoxicity was evaluated.

The animals were sacrificed using sodium pentobarbital anesthesia. Heart tissues were snap frozen in liquid nitrogen for gene and protein expression analyses or fixed with 4% paraformaldehyde for histological analysis.

### Cardiac Function Evaluation

Cardiac function was determined non-invasively by transthoracic echocardiography in anesthetized mice 1 day before sacrifice as described previously (Kandalam et al., 2010). The mice were anesthetized using isoflurane, and echocardiography was performed with a SONOS 5500 ultrasound (Philips Electronics, Amsterdam, Netherland) with a 15-MHz linear array ultrasound transducer.

### Real-Time PCR

RNA was isolated from cultured H9C2 cells and heart tissue by using TRIZOL (Thermo Fisher Scientific, United States). Reverse transcription and quantitative PCR were carried out using a two-step PrimeScript^TM^ RT reagent kit (Perfect Real Time) (TAKARA), Eppendorf Mastercycler ep realplex detection system (Eppendorf, Hamburg, Germany) for reverse transcription and QuantStudio3 Real-Time PCR Systems (Applied Biosystems, Thermo Fisher Scientific, United States) for real-time PCR. Primers for the genes were synthesized and obtained from Thermo Fisher Scientific. The primer sequences are presented in Supplementary Table 1. mRNA levels of the target genes were normalized to *Actb* gene mRNA.

### Western Blot Analysis

Fifty micrograms of total protein from cell or tissue lysates was separated by 10% SDS-PAGE and electrotransferred to PVDF membranes. Membranes were blocked in Tris-buffered saline containing 0.05% Tween 20 and 5% non-fat milk for 1.5 h. The PVDF membranes were then incubated with specific primary antibodies. Immunoreactive bands were detected by incubating the membranes with secondary antibodies conjugated to horseradish peroxidase and an enhanced chemiluminescence reagent (Bio-Rad). Densitometric quantification was performed using ImageJ analysis software version 1.38e and normalized to their respective controls (glyceraldehyde 3-phosphate dehydrogenase (GAPDH) for cytosolic proteins, Lamin B for nuclear fractions, and total protein for phosphorylated-form detection).

### Heart Histology and Immunostaining

Hearts were fixed in 4% paraformaldehyde and embedded in paraffin. Five-micrometer-thick sections were stained with hematoxylin and eosin (H&E) (C0105, Beyotime Biotechnology) for histological analysis and Sirius Red and Masson’s trichrome (G1340-7 × 100 ml, Solarbio Life Science) to evaluate cardiac fibrosis. The sections were observed under a light microscope (Nikon, Japan).

For immunohistochemical staining, the sections were deparaffinized and rehydrated. The sections were treated with 3% H2O2 for 30 min to block endogenous peroxidase activity and then with 1% BSA in PBS for 30 min. The slides were incubated overnight at 4°C with the primary antibody (TNF-α, 1:50; both from Santa Cruz). Peroxidase-conjugated secondary antibodies were used for detection (Santa Cruz; 1:100 dilution; 1 h incubation). The slides were counterstained with hematoxylin for 5 min, dehydrated, and mounted. Images were viewed by a bright field microscope (Nikon).

### ELISA

The TNF-α (70-EK382/3-96) levels in heart tissue were measured using ELISA kits (eBioscience, San Diego, CA, United States). All experiments followed the manufacturer’s instructions.

### siRNA Transfection and Gene Silencing

Gene silencing in cells was achieved using specific siRNA sequences. TLR2 siRNAs were purchased from GenePharma Co., Ltd. (Shanghai, China). Custom siRNAs were synthesized for rat TLR2, TLR4, and MD2. The sequences are presented in Supplementary Table 1. The H9C2 cells were transfected with siRNA using LipofectAMINE^TM^ 2000 (Thermo Fisher Scientific, Carlsbad, CA, United States).

### Immunoprecipitation

Following treatments, cell lysates or heart tissues were prepared and incubated with an anti-TLR2 or MyD88 antibody overnight. Complexes were retrieved with protein G-Sepharose beads at 4°C for 4 h. The TLR2 and MyD88 levels were further detected by immunoblot using anti-TLR2 and MyD88 antibodies (IB), respectively.

### Computational Docking and Molecular Simulation

The crystal structure of the TIR domain of human TLR2 (PDB code 1FYW) was derived from Protein Data Bank repository. Input files of ligand and receptor for docking were prepared using Graphical User Interface program AutoDock Tools 1.5.6 (The Scripps Research Institute, La Jolla, CA, United States) (Morris et al., 2009). Molecular docking was performed by AutoDock Vina 1.0.2. Celastrol was docked into the TIR domain of TLR2 to generate 20 binding poses, respectively. The binding free energy between each docking pose and TLR2 was scored by the MM/GBSA method in AmberTools package after a structure minimization (Salomon-Ferrer et al., 2012). Finally, based on the per-residue decomposition energy calculations, the key residues for protein-ligand interaction were identified.

### Statistical Analysis

The data presented in this study are representative of five independent experiments and are expressed as the mean ± SEM. The exact group size (n) for each experiment is provided, and “n” refers to independent values. Statistical analysis was performed with GraphPad Prism 8.0 software (San Diego, CA, United States). We used one-way ANOVA followed by Dunnett’s *post hoc* test when comparing more than two groups of data and one-way ANOVA and non-parametric Kruskal–Wallis tests followed by Dunn’s *post hoc* test when comparing multiple independent groups. A *P* value < 0.05 was considered to be statistically significant. Post-tests were run only if F achieved *P* < 0.05 and there was no significant variance in homogeneity.

## Results

### LCZ696 Treatment and TLR2 Deficiency Attenuated Doxorubicin-Induced Cardiac Systolic Dysfunction

Firstly, our objective was to determine whether LCZ696 and TLR2 deficiency could inhibit the doxorubicin-induced cardiotoxicity in mice. As previously reported, doxorubicin was used to establish chronic cardiac injury in mice through intraperitoneal injection. Then, LCZ696 was administered orally to determine whether it could prevent heart injury, and the model of doxorubicin-induced cardiac injury was also established in TLR2KO mice to explore the potential role of TLR2.

**FIGURE 1:**
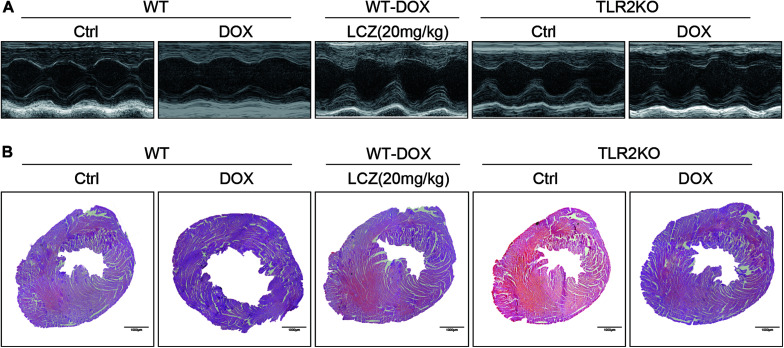
LCZ696 treatment and TLR2 deficiency attenuated doxorubicin-challenged systolic dysfunction. **(A)** Representative echocardiogram images of each group. **(B)** Representative H&E staining of heart tissues showing the effect of LCZ696 and TLR2 deficiency on doxorubicin-induced dilated cardiomyopathy in mice [original magnification 1×].

Before sacrificed, the cardiac function of each group was evaluated by external echocardiography (Figure 1A and Table 1). As shown in Figure 1A and Table 1, DOX significantly impaired heart function by decreasing EF%, FS%, IVSD, and PWd and increasing LVIDd, which resulted in serious systolic dysfunction. Interestingly, these challenges were normalized with oral LCZ696 treatment and TLR2 deficiency, which indicated that LCZ696 and TLR2 deficiency attenuated DOX-induced cardiac systolic dysfunction in mice.

**TABLE 1:** Biometric and echocardiographic parameters of the experimental mice.

	WT			TLR2KO	
	Ctrl *N* = 7	DOX *N* = 7	DOX + LCZ *N* = 7	Ctrl *N* = 7	DOX *N* = 7
EF%	80.14 ± 1.05	75.74 ± 1.08***	78.25 ± 1.17^###^	81.08 ± 1.75	79.18 ± 2.01^###^
FS%	40.26 ± 2.49	35.11 ± 2.11***	38.36 ± 2.47^###^	39.27 ± 1.81	38.75 ± 1.41^###^
LVIDd, mm	2.11 ± 0.28	2.35 ± 0.56**	2.18 ± 0.14^##^	2.10 ± 0.58	2.15 ± 0.78^##^
IVSD, mm	0.98 ± 0.11	0.95 ± 0.14*	0.99 ± 0.22^#^	0.98 ± 0.07	0.99 ± 0.1^#^
PWd, mm	0.70 ± 0.03	0.67 ± 0.04**	0.71 ± 0.13^#^	0.71 ± 0.21	0.71 ± 0.35^#^
E wave, m/s	0.68 ± 0.05	0.70 ± 0.07**	0.69 ± 0.07^#^	0.67 ± 0.08	0.69 ± 0.11^#^
Tei Index	0.82 ± 0.04	0.80 ± 0.10**	0.82 ± 0.12^#^	0.81 ± 0.11	0.82 ± 0.25^#^
IRT, ms	15.33 ± 1.14	17.28 ± 1.58*	14.56 ± 1.97^#^	14.23 ± 1.46	14.28 ± 1.41^#^

Transthoracic echocardiography was performed on mice at the end of the animal study. EF, ejection fraction%, FS, fractional shortening%; LVIDd, diastole left ventricle internal dimension; PWd, diastole posterior wall thickness; IVSd, diastole interventricular septal thickness; E wave; IRT, isovolumic relaxation time; Tei index; Data are presented as the mean ± SEM, *n* = 7. *P < 0.05 compared to ctrl; **P < 0.01 compared to ctrl; ***P < 0.001 compared to ctrl; #*P* < 0.05 compared to Ang II; ##*P* < 0.01 compared to Ang II; ###*P* < 0.001 compared to Ang II; ns = not significant.

To deeply explore the alterations of the heart cavity, histological assessments of the whole heart were performed for all groups by H&E staining. As shown in Figure 1B, DOX induced thinning of the ventricular wall and enlargement of the heart cavity, which were obviously improved in the hearts of LCZ696-treated and TLR2-deficient mice. These above results show that treatment with LCZ696 and TLR2 deficiency prevented DOX-induced cardiac systolic dysfunction.

### LCZ696 Treatment and TLR2 Deficiency Alleviated Doxorubicin-Induced Cardiac Fibrosis

**FIGURE 2:**
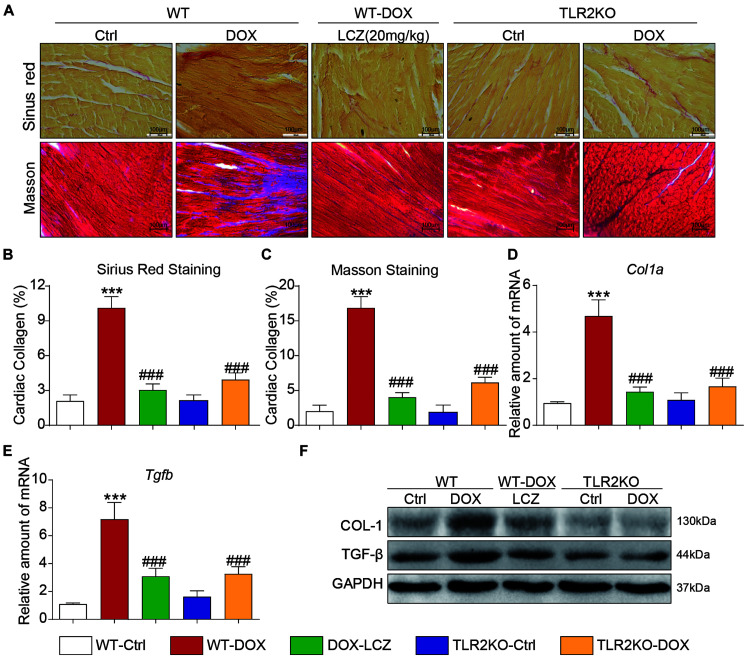
LCZ696 treatment and TLR2 deficiency attenuated doxorubicin-induced cardiac fibrosis. **(A)** Representative images of Sirius Red and Masson trichrome staining of longitudinal sections of the hearts (200×). **(B,C)** Quantification of interstitial fibrotic areas (%) from Sirius Red-stained heart sections **(B)** and Masson’s Trichome staining **(C)**. **(D,E)** The mRNA levels of *Col1a1* **(D)** and *Tgfb* **(E)** were detected by real-time PCR in the heart tissue from the above transplanted mice (data were normalized to *Actb*). **(F)** Heart tissue from each group of mice was homogenized for western blot analysis of collagen I, TGF-β, and GAPDH. (*n* = 7; *** *P* < 0.001 as indicated; ###*P* < 0.001 compared to Ang II; and ns is not significant).

Next, we assessed fibrosis in the heart tissues. Masson’s trichrome and Sirius Red stains were chosen to evaluate connective tissue and collagen, respectively, and validated the anti-fibrotic effects of LCZ696 and TLR2 deficiency. As shown in Figures 2A–C, DOX promoted collagen deposition in myocardial tissue, and these histological changes were obviously improved in LCZ696-treated and TLR2-deficient mice.

In addition to the histological results, the hearts from DOX-challenged mice showed significantly increased mRNA levels of the profibrotic genes *Col1a* (Figure 2D) and *Tgfb* (Figure 2E), which were notably reduced by LCZ696 and TLR2 deficiency. These results paralleled the protein levels of collagen I (COL-I) and TGF-β in the hearts of each group (Figure 2F and Supplementary Figures 1A,B).

These results demonstrated that LCZ696 treatment and TLR2 deficiency reduced doxorubicin-induced cardiac fibrosis.

### LCZ696 Treatment and TLR2 Knockdown Attenuated Doxorubicin-Induced Cardiac Inflammation in Mice

As previously reported, fibrosis and inflammation complement each other during diseases. Moreover, considering the potential effect of LCZ696 and the critical role of TLR2 in anti-inflammatory processes, we next assessed the inflammatory response in the hearts of each group.

**FIGURE 3:**
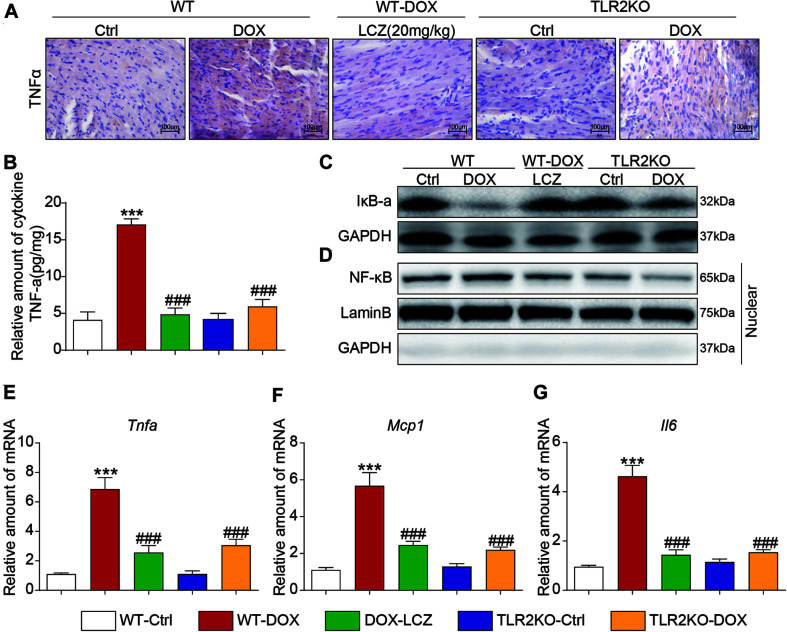
LCZ696 treatment and TLR2 knockdown attenuated doxorubicin-induced cardiac inflammation in mice. **(A)** Representative images of anti-TNF-α staining in the hearts of each group (200×). **(B)** TNF-α levels in mouse heart tissue homogenates determined by ELISA. **(C)** Western blot analysis of IκBα levels in heart tissue. GAPDH was used as a loading control. **(D)** Nuclei were isolated from mouse heart tissue, and NF-κB in the nucleus was detected by western blot. Lamin B and GAPDH were used as controls. **(E–G)** Real-time PCR was used to determine the mRNA levels of *Tnfa* **(E)**, *Mcp1* **(F)**, and *Il-6* **(G)** in mouse heart tissue (data were normalized to *Actb*). (*n* = 7; *** *P* < 0.001 vs. WT control group; ### *P* < 0.001 vs. DOX group).

Immunohistochemistry staining showed that TNF-α was significantly increased in the DOX treated mice compared to the control mice. As expected, LCZ696 treatment and TLR2 knockdown prevented the heart from developing high TNF-α expression (Figure 3A and Supplementary Figure 2A). Similar results were also observed in the ELISA results (Figure 3B). NF-κB-related proteins, as the well-established downstream signaling proteins in the TLR2 pathway, participate in acute and chronic inflammation. Then, we tested whether these alterations occurred in heart tissues. As shown in Figures 3C,D, LCZ696 treatment and TLR2 deficiency effectively prevented IκBα degradation (Figure 3C) and inhibited the nuclear translation of NF-κB (Figure 3D) in heart tissue compared with the DOX-induced mice. Moreover, the mRNA levels of the pro-inflammation-related genes *Tnf*α (Figure 3E), monocyte chemotactic protein 1 (*Mcp1*) (Figure 3F), and *Il6* (Figure 3G) were validated by RT-qPCR, which provided additional evidence to show the anti-inflammatory effects of LCZ696 treatment and TLR2 deficiency.

Thus, these results indicated that LCZ696 treatment and TLR2 deficiency significantly inhibited DOX-induced cardiac inflammation in heart tissues, which may be associated with the cardioprotective effect.

### LCZ696 Treatment and TLR2 Knockdown Attenuated Doxorubicin-Induced H9C2 Cell Fibrosis *in vitro*

Next, H9C2 cells were cultured to further confirm the potential effect of LCZ696 treatment and TLR2 deficiency in cardiac injury induced by DOX, and we then determined the role of TLR2 in DOX treatment.

**FIGURE 4:**
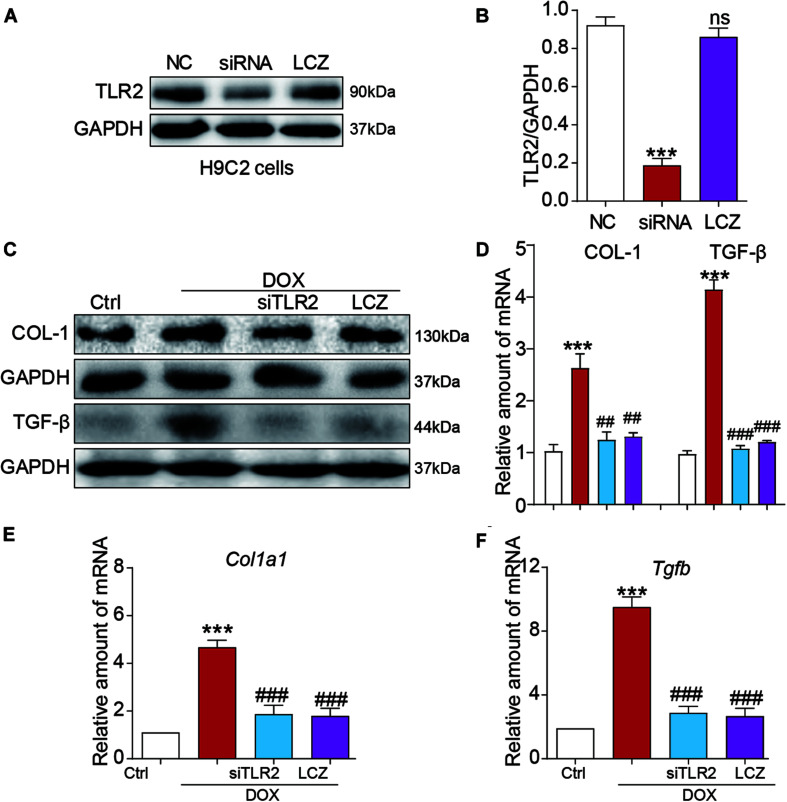
LCZ696 treatment and TLR2 knockdown attenuated doxorubicin-induced H9C2 cell fibrosis *in vitro*. **(A)** H9C2 cells were transfected with control siRNA (NC) or TLR2 siRNA (siTLR2) and treated with LCZ696 (20 μmol/l) for 24 h. Western blot detected TLR2 and GAPDH protein levels. **(B)** Densitometric quantification of TLR2/GAPDH levels in the immunoblots presented in panel **(A)**. **(C)** H9C2 cells were transfected with control siRNA or TLR2 siRNA and pre-treated with LCZ696 (20 μmol/l) and then stimulated with DOX (1 μM) for 24 h. Western blot analysis of the protein levels of collagen I and TGF-β in whole cell lysates. **(D)** Densitometric quantification of collagen I/GAPDH and TGF-β/GAPDH levels in immunoblots presented in panel **(C)**. **(E,F)** The mRNA levels of *Col1a1* **(E)** and *Tgfb* **(F)** were detected by real-time PCR. (*n* = 5; *** *P* < 0.001 vs. Ctrl group; ##*P* < 0.01 compared to Ang II; ### *P* < 0.001 vs. DOX group).

Toll-like receptor 2 siRNA was used to silence the expression of TLR2 in the H9C2 cell line (Figures 4A,B). As expected, DOX stimulated significantly high protein levels of COL-I and TGF-β protein as well as the mRNA levels, which were normalized to the control levels as the TLR2 was knocked down or pre-treated with LCZ696 in H9C2 cells (Figures 4C–F).

### LCZ696 Treatment and TLR2 Knockdown Attenuated the Doxorubicin-Induced H9C2 Cell Inflammatory Response *in vitro*

**FIGURE 5:**
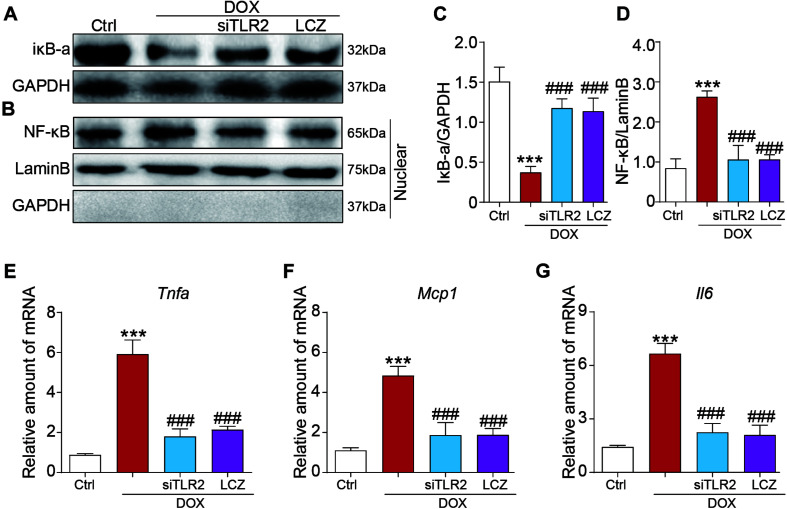
LCZ696 treatment and TLR2 knockdown attenuated the doxorubicin-induced H9C2 cell inflammatory response *in vitro*. **(A,B)** H9C2 cells were transfected with control siRNA, TLR2 siRNA, and LCZ696 (20 μmol/l) and then stimulated with DOX (1 μM) for 8 h. Western blot detected IκBa and GAPDH protein levels in whole cell lysates **(A)** and detected NF-κB, Lamin B, and GAPDH protein levels in the nuclear extractions **(B)**. **(C)** Densitometric quantification of IκBa/GAPDH levels in the immunoblots presented in panel **(A)**. **(D)** Densitometric quantification of NF-κB/Lamin B levels in the immunoblots presented in panel **(B)**. **(E–G)** The mRNA levels of *Tnfa* **(E)**, *Mcp1* **(F)**, and *Il-6* **(G)** were measured by real-time PCR. (*n* = 5; *** *P* < 0.001 vs. Ctrl group; ### *P* < 0.001 vs. DOX group).

Furthermore, we also assessed the inflammatory response induced by DOX *in vitro*. Similar to the above results, TLR2 silencing or pre-treated with LCZ696 significantly reversed DOX-induced IκB-a degradation and the nuclear translocation of NF-κB (Figures 5A–D). In addition, cardiac inflammation was ameliorated by TLR2 knockdown or LCZ696 treatment, as shown by the gene expression of *Tnfa*, *Mcp1*, and *Il-6* (Figures 5E–G).

These results provide evidence that LCZ696 treatment and TLR2 knockdown attenuated doxorubicin-induced cardiac cell fibrosis and inflammation *in vitro*.

### Administration of LCZ696 Attenuated Doxorubicin-Induced Cardiac Injury by Inhibiting TLR2-MyD88 Complex Formation

To explore the underlying mechanism of the effect of LCZ696 and TLR2 deficiency in DOX-related cardiac injury, we analyzed the TLR2-MyD88 complex formation and the well-established TLR2 downstream pathway.

**FIGURE 6:**
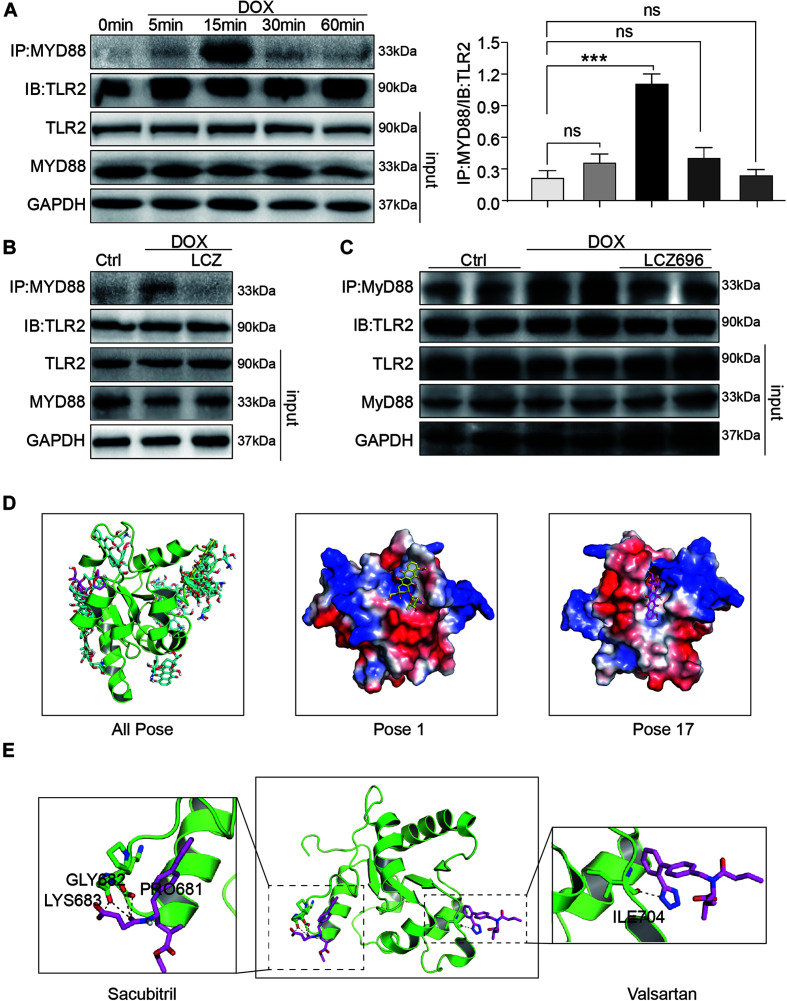
LCZ696 treatment attenuated doxorubicin-induced cardiac injury by inhibiting TLR2-MyD88 complex formation. **(A)** Time course of the DOX-induced TLR2-MyD88 interaction in H9C2 cells. Cells were treated with 1 μM DOX for up to 1 h. Quantification is shown below. **(B)** H9C2 cells were pre-treated with LCZ696 (20 μmol/l) for 1 h and then stimulated with DOX (1 μM) for 30 min. The TLR2-MyD88 interaction was analyzed. **(C)** A co-immunoprecipitation assay determined the TLR2-MyD88 interaction in mice heart tissues. **(D)** The total of 20 binding conformations (right) are produced by the docking program QVina-W, of which pose 1 (marked yellow) has the best docking score. The details of the binding pose of DOX with the lowest binding energy for TLR2-TIR is shown in the middle, and the details of the binding pose of DOX with TLR2-TIR calculated by MM/GBSA method is shown in the right. **(E)** The details of the binding pose of LCZ696 (Sacubitril-right and Valsartan-left) with the lowest binding energy is shown for TLR2-TIR. Magenta is the optimal conformation for scoring (unit is kcal/mol). ***P < 0.001 compared to ctrl.

Firstly, H9C2 cells were treated with DOX in a time-dependent manner. The Co-IP assay results showed that DOX significantly increased the TLR2-MyD88 interaction in 15 min (Figure 6A), which indicated the potential mechanism of TLR2-mediated DOX-related cardiomyopathy. More interestingly, DOX-induced TLR2-MyD88 complex formation was attenuated by pre-treatment with LCZ696 (Figure 6B). Besides, we further tested the effect of LCZ696 on TLR2-MyD88 complex formation in mice heart tissues. As the data shown in Figure 6C, with no alteration in TLR2 and Myd88 protein level, DOX significantly promoted TLR2-MyD88 interaction in the mouse heart evidenced by Co-IP assay and treated with LCZ696 attenuated the formation of TLR2-MyD88 complex. These results indicated that the treatment effect of LCZ696 manifested by inhibiting the TLR2-MyD88 complex formation induced by DOX.

To understand the potential molecular mechanism of DOX interacting with TLR2 protein, we conducted a molecular docking and simulation study using the crystal structure of the TIR domain of human TLR2 (PDB code 1FYW). The TIR domain, as the most important structures of TLR2, is responsible for MyD88 interaction and the activation of downstream inflammation signaling pathway (O’Neill and Bowie, 2007).

The docking uses the QVina-W program to search in the global scope of the protein surface. As shown in the Figure 6D, a total of 20 binding conformations are generated, among which pose1 (marked in yellow) has the best conformation score for docking.

To predict the binding position of DOX on TIR domain, a per-residue decomposition energy calculation was performed for the 20 docking poses (Supplementary Figure 4).

Distributions of both docking scores and MM/GBSA scores show that DOX may bind the TIR domains of TLR2 (Figure 6D, right). Greater negative scores are obtained in DOX- TIR domain interaction, indicating that DOX has a higher binding affinity to TIR domain in pose17compared to the pose1. Five key residues in the TIR domain with the top-lowest average energy values are Glu-727, Ile-686, Arg-677, Trp-684, and Pro-681 (Figure 6D and Supplementary Figure 4). Among these, ARG-677 showed the lowest energy. Among these, based on the energy values, Pro-681 and Glu-727 appeared to be key residues which DOX combined with TLR2.

Similarly, we also conducted the molecular docking and simulation study of TLR2 and LCZ696. The pose in Figure 6E is the best conformation of 20 top-scoring docking conformations of LCZ696 and TLR2 (Supplementary Figure 5). As shown in Figure 6E, the key residues in the Sacubitril with the top-lowest average energy values are Pro-681, Gly-682, and Lys-683 (Figure 6E). And for the Valsartan, Ser-704 showed the lowest energy (Figure 6E). Among these, Pro-681 and Ser-704 appeared to be key residues based on the energy values, which predicts the potential mechanism by which LCZ696 affects the interaction between DOX and TLR2.

Furthermore, we found that the TLR2-MyD88 complex formation induced by DOX was independent with TLR4 and MD2, as it was not affected by TLR4 or MD2 knockdown (Supplementary Figures 6A,B).

**FIGURE 7:**
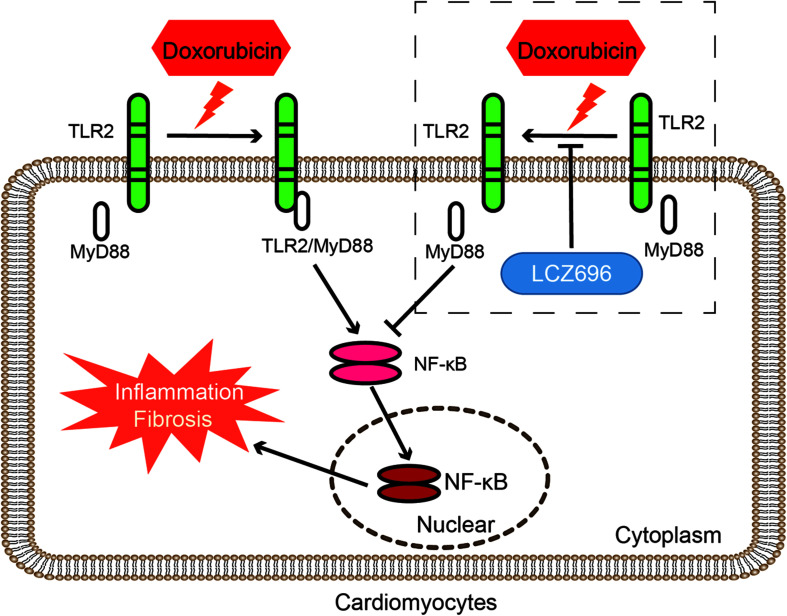
LCZ696 attenuated doxorubicin-induced heart injury through the TLR2-MyD88 pathway. The mechanism by which LCZ696 relieves DOX-induced cardiac inflammation, fibrosis and heart failure lies in reducing the formation of TLR2-MyD88 complexes.

In conclusion, our results found that the mechanism by which LCZ696 relieves DOX-induced cardiac injury lies in inhibiting the formation of TLR2-MyD88 complexes (Figure 7).

## Discussion

Dilated cardiomyopathy is one of the most serious side effects of doxorubicin treatment, significantly reducing its anticancer value and causing a large societal burden. However, the mechanisms of DCM are still unclear, and an effective therapy to prevent the progression of existing cardiac inflammation and fibrosis induced by DOX are urgently needed. Our study aimed to determine the potential effect of LCZ696 and the role of TLR2 in DOX-induced cardiac dysfunction, fibrosis and inflammation, which may help to find an effective drug to protect the heart and provide a potential signaling pathway for the treatment of DOX-induced DCM.

In our study, we showed that LCZ696 treatment and TLR2 deficiency attenuated DOX-related dilated HF by improving the decreased EF% and increased LVIDd. In addition, *in vivo* and *in vitro*, our results showed that DOX stimulated the expression of matrix proteins and inflammatory cytokines in the heart and that LCZ696 treatment and TLR2 knockdown ameliorated these alterations and ultimately improved cardiac function. Furthermore, the underlying mechanisms involved in DOX-induced cardiomyopathy were revealed that DOX stimulated the formation of the TLR2-MyD88 complex, which activated the NF-κB pathway, leading to cardiac cell inflammation and fibrosis. This TLR2-MyD88 interaction was TLR4 or MD2 independent and could be inhibited by LZC696, which explained the strong effect of this drug in preventing heart injury caused by DOX.

It is well established that LCZ696, as a novel angiotensin receptor-neprilysin inhibitor, significantly reduced mortality and hospitalization due to heart failure in HF patients with a reduced ejection fraction (HFrEF) (Mann et al., 2020). The results of the PARADIGM-HF trial (McMurray et al., 2014) suggested a second function of sacubitril/valsartan: degrading peptides that regulate the cardiovascular, nervous, inflammatory, and immune systems (Turner et al., 2001; D’Elia et al., 2017). Recent studies found that LCZ696 increased local BNP/CNP levels, interfered with angiotensin II-mediated signaling, and then reduced the magnitude of cardiac remodeling in animal models of hypertension and myocardial infarction (von Lueder et al., 2015; Oatmen et al., 2018). Regarding the cardiotoxicity induced by DOX, accumulating evidences have discovered the pathophysiological mechanisms, but the treatments to mitigate cardiac damage are still limited (Hu et al., 2020; Yang et al., 2020). Xia et al. (2017) found that Drp1 and its Ser-616 phosphorylation were significantly increased in DCM patients and demonstrated that the use of LCZ696 against DOX-induced cardiac dysfunction is associated with alleviated Drp1-mediated mitochondrial dysfunction. Similar to our results, we focused on the anti-inflammatory effect of LCZ696. Our data indicated that LCZ696 prevented IκBα degradation, inhibited the nuclear translation of NF-κB and reduced the expression of inflammatory cytokines *in vivo* and *in vitro*. More impressively, we found that pre-treatment with LCZ696 inhibited the increased formation of the TLR2/MyD88 complex induced by DOX. This finding partly explains the mechanism of the anti-inflammatory effect of LZC696.

Similar to other TLRs, TLR2 is the most characteristic member of pattern recognition receptors (PRRs), which play an important role in innate immune mechanisms (Jacquet and Robinson, 2020). TLRs play different roles in different stages of infection of atherosclerosis-related pathogens such as Chlamydia pneumoniae (Li B. et al., 2020). Since Nozaki et al. (2004) found that TLR2 may play a role in the regulation of inflammatory and apoptotic mediators in the heart after DOX administration in 2004, little research has explored the mechanism by which TLR2 mediates DOX-induced cardiotoxicity. Our current study indicated that DOX induced the increased formation of the TLR2/MyD88 complex, which leads to the activation of the NF-κB pathway and stimulates the expression of cardiac inflammation and fibrosis. This result partially compensates for the inflammatory mechanism of DOX-induced myocardial injury. Moreover, we found that this interaction of TLR2 and MyD88 induced by DOX is independent with TLR4 or MD2, but the clear role of TLR4 and MD2 in DOX-induced cardiac side effects still needs further research. Finally, we also performed the molecular docking and simulation study to find potential key residues where DOX combined with to stimulating the interaction of TLR2 and MyD88, and which prevented from by LCZ696. We were careful in our interpretation and reporting of the data and elected not to overstate that these residues are the specific positions of the cardiotoxicity of DOX and the cardioprotection of LCZ696 in DOX induced cardiac failure.

Taken together, our results demonstrated that the mechanism by which LCZ696 relieves DOX-induced cardiac inflammation fibrosis and HF lies in reducing the formation of TLR2-MyD88 complex. LCZ696 may be a potential drug to treat DOX-related HF, and TLR2-MyD88 could be a parallel target in the prevention and treatment of DOX-related heart injury.

## Data Availability Statement

The raw data supporting the conclusions of this article will be made available by the authors, without undue reservation.

## Ethics Statement

The animal study was reviewed and approved by the Animal Policy and Welfare Committee of Wenzhou Medical University (Approval Document No. wydw2016-0124).

## Author Contributions

SY, LS, PS, BY, and GL: conception and design, collection, analysis, and interpretation of data, and manuscript writing. SY, LS, and SW: collection, interpretation, and analysis of data. GL and WH: conception and design, interpretation of data, and manuscript revision. All authors contributed to the article and approved the submitted version.

## Conflict of Interest

The authors declare that the research was conducted in the absence of any commercial or financial relationships that could be construed as a potential conflict of interest.

## Footnotes

1 http://www.gpower.hhu.de/

## Abbreviations

COL-I: collagen I
DMSO: dimethyl sulfoxide
DOX: doxorubicin
GAPDH: glyceraldehyde 3-phosphate dehydrogenase
HF: heart failure
IL-6: interleukin 6
MCP-1: monocyte chemotactic protein 1
NC: negative control
NF- κ B: nuclear factor- κ B
TGF- β: transforming growth factor- β
TLR: toll-like receptor
TNF- α: tumor necrosis factor- α.
