# Supplementary material for: LCZ696 Attenuated Doxorubicin-Induced Chronic Cardiomyopathy Through the TLR2-MyD88 Complex Formation
Source: Front Cell Dev Biol. 2021 Apr 13;9:654051. doi: 10.3389/fcell.2021.654051 (PMC8076895; doi:10.3389/fcell.2021.654051)
Supplement: Supplementary file 1 [file Data_Sheet_1.docx]

***Supporting Information***

**LCZ696 attenuated doxorubicin-induced chronic cardiomyopathy through the TLR2-MyD88 complex formation**

Shiju Ye^1,2,3#^, Lan Su^1,2,#^, Peiren Shan^1,2^, Bozhi Ye^1,2^, Shengjie Wu^1,2^, Guang Liang^1,3*^,Weijian Huang^1,2*^

*1 Department of Cardiology, the First Affiliated Hospital of Wenzhou Medical University, Wenzhou, China.*

*2 The Key Lab of Cardiovascular Disease of Wenzhou, Wenzhou, China.*

*3 Chemical Biology Research Center, School of Pharmaceutical Sciences, Wenzhou Medical University, Wenzhou, Zhejiang, China.*

# These authors contribute equally to this paper.

Corresponding author: Weijian Huang; Co-corresponding author: Guang Liang

**Short Title:** LCZ696 attenuated DOX-induced cardiac injury through TLR2

**Supplemental Table 1.** Primer sequences for qPCR and siRNA.

| **Gene** | **Species** | **Sequence** |
| --- | --- | --- |
| *Col1a* | Rat | GACATCCCTGAAGTCAGCTGC  TCCCTTGGGTCCCTCGAC |
| *Tgfb* | Rat | GCAACAACGCAATCTATGAC  CCTGTATTCCGTCTCCTT |
| *Actb* | Rat | AAGTCCCTCACCCTCCCAAAAG  AAGCAATGCTGTCACCTTCCC |
| *Tnfa* | Rat | AGGAGGAATTTGGCCAGGTG  GCTCACGAGGAGGCTAATCC |
| *Mcp1* | Rat | GTCACCAAGCTCAAGAGAGAGA  GAGTGGATGCATTAGCTTCAGA |
| *Il6* | Rat | GAGTTGTGCAATGGCAATTC  ACTCCAGAAGACCAGAGCAG |
| *Tgfb* | Mouse | TGACGTCACTGGAGTTGTACGG  GGTTCATGTCATGGATGGTGC |
| *Col1a* | Mouse | TGGCCTTGGAGGAAACTTTG  CTTGGAAACCTTGTGGACCAG |
| *Tnfa* | Mouse | TGATCCGCGACGTGGAA ACCGCCTGGAGTTCTGGAA |
| *Mcp1* | Mouse | TCACCTGCTGCTACTCATTCACCA  TACAGCTTCTTTGGGACACCTGCT |
| *Il6* | Mouse | GAGGATACCACTCCCAACAGACC AAGTGCATCATCGTTGTTCATACA |
| *Actb* | Mouse | CCGTGAAAAGATGACCCAGA  TACGACCAGAGGCATACAG |
| siRNA-TLR2 | Rat | GCAGGUGACAACCAUUUCATT  UGAAAUGGUUGUCACCUGCTT |
| siRNA-TLR4 | Rat | GGCUCAUAAUCUUAUACAUTT AUGUAUAAGAUUAUGAGAGCCTT |
| siRNA-MD2 | Rat | CGAAGCGCAAGGAAAUUGUTT  ACAAUUUCCUUGCGCUUCGTT |


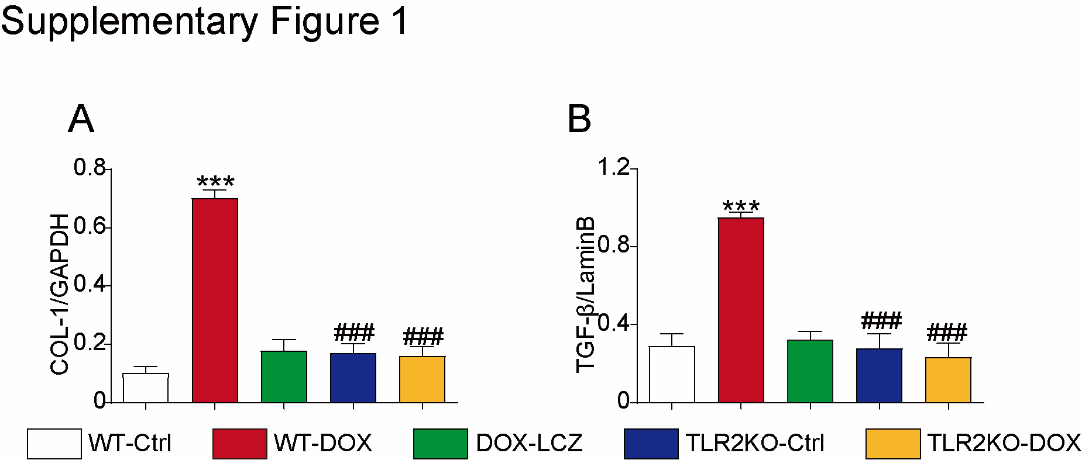


**Supplemental Figure 1:** Heart tissue lysates were prepared and probed for collagen I (A) and TGF-β (B). Densitometric quantification of Figure 2F presented here is normalized to GAPDH (n=6-9; * P<0.05, ** P<0.01, *** P<0.001, vs Ctrl group; # P<0.05, ## P<0.01, ### P<0.001, vs DOX group).


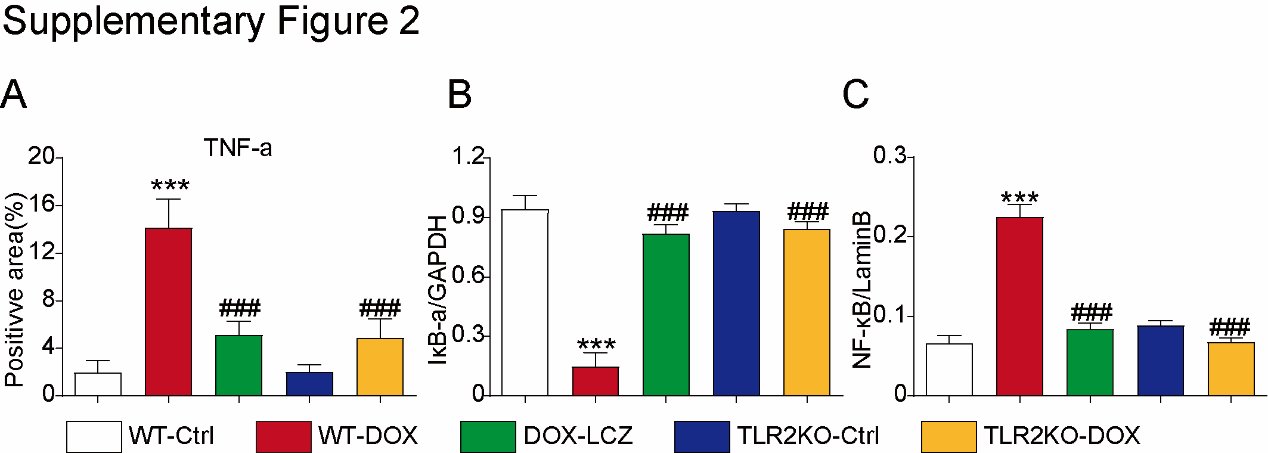


**Supplemental Figure 2:** (A) Quantification of interstitial TNF-α-positive areas (%) from heart sections. Densitometric quantification of the data is shown in Figure 3A. (B-C) Heart tissue lysates were prepared and probed for iκB-a (B) in heart tissues and NF-κB (C) in nuclear extracts prepared from mouse heart tissues. The densitometric quantification presented here was normalized to GAPDH (Figure 3C) and Lamin B (Figure 3D). (n=7; * P<0.05, ** P<0.01, *** P<0.001, vs Ctrl group; # P<0.05, ## P<0.01, ### P<0.001, vs DOX group).


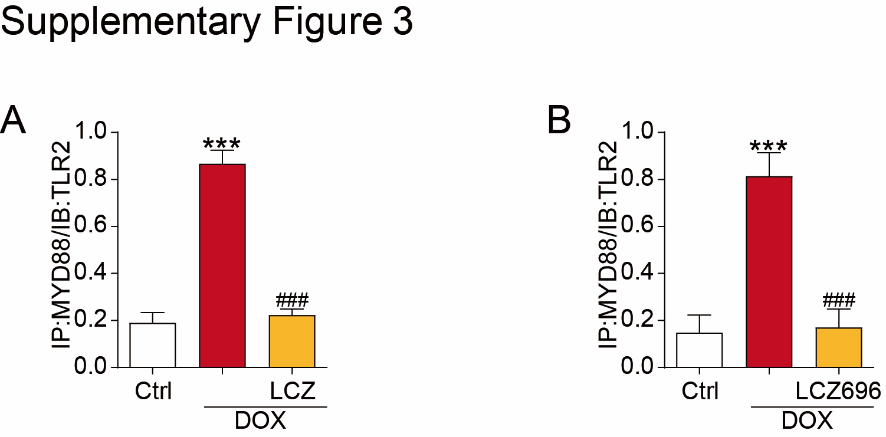


**Supplemental Figure 3:** (A) H9C2 cells were prepared and probed for TLR2-MyD88 complex formation. Densitometric quantification of the data is shown in Figure 6B. (n=3; * P<0.05, ** P<0.01, *** P<0.001, vs Ctrl group; # P<0.05, ## P<0.01, ### P<0.001, vs DOX group); (B) Heart tissue lysates were prepared and probed for TLR2-MyD88 complex formation. Densitometric quantification of the data is shown in Figure 6C. (n=6-9; * P<0.05, ** P<0.01, *** P<0.001, vs Ctrl group; # P<0.05, ## P<0.01, ### P<0.001, vs DOX group).


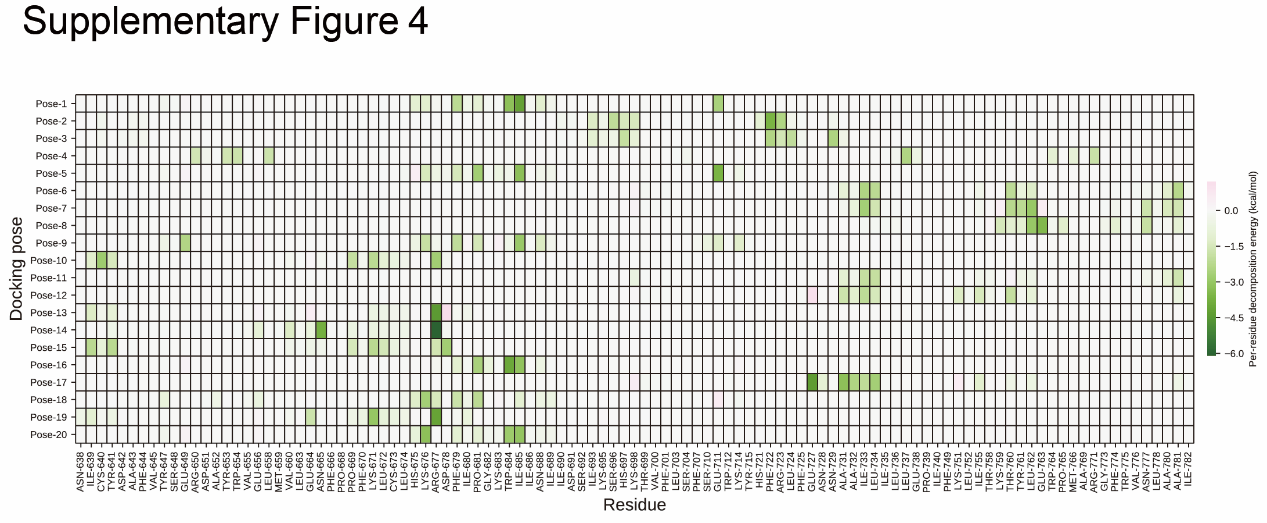


**Supplemental Figure 4:** The heat map of per-residue decomposition energy (kcal/mol) of all the 20 poses calculated by the MM/GBSA method (the darker the green, the greater the contribution).


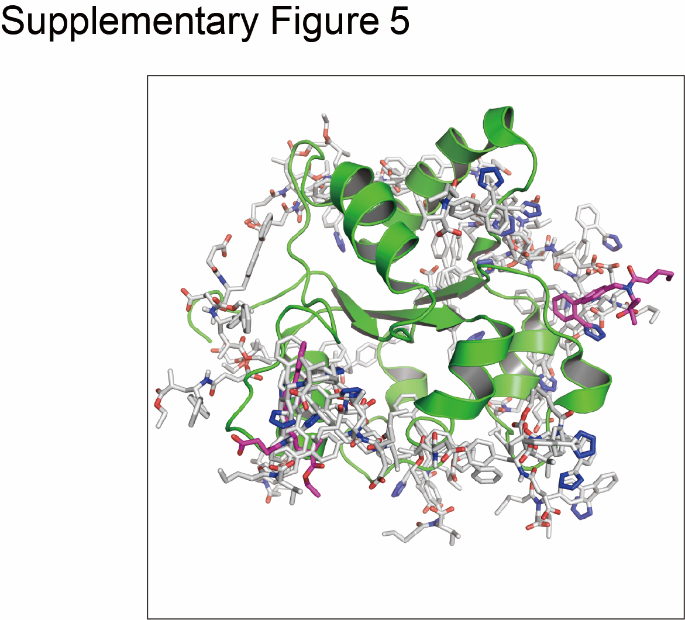


**Supplemental Figure 5:** 20 top-scoring docking conformations of LCZ696 and TLR2, of which magenta is the best conformation (kcal/mol)


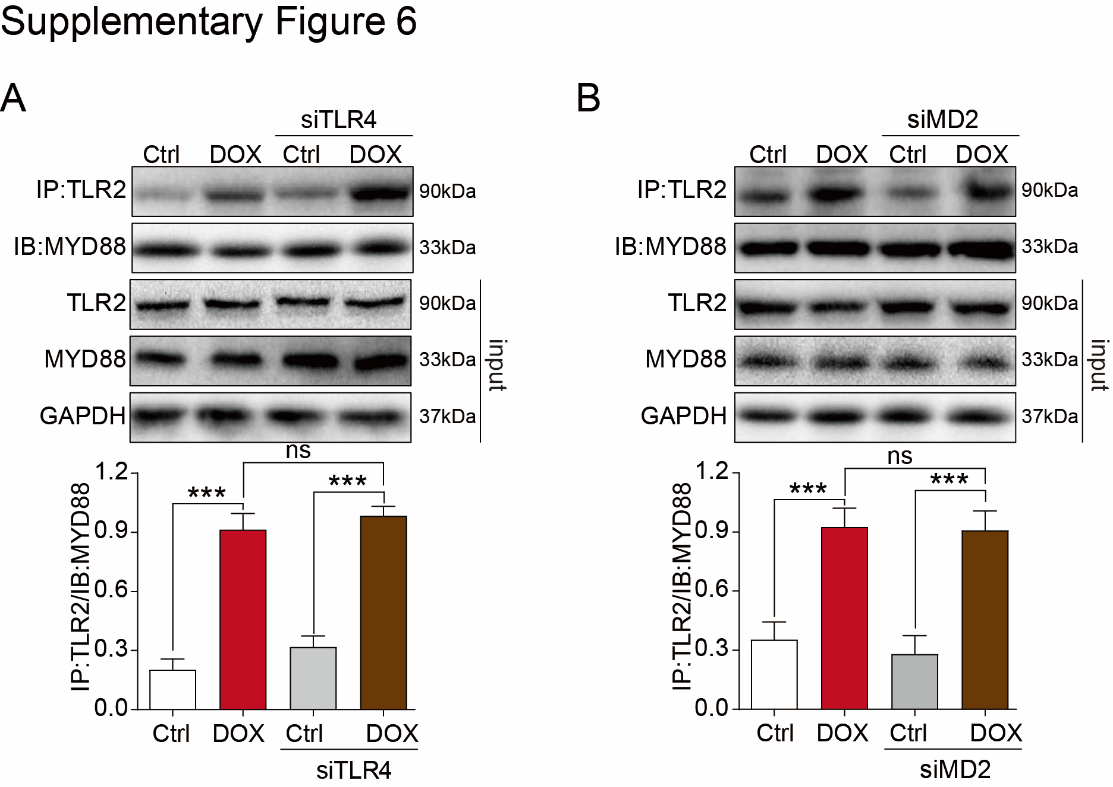


**Supplemental Figure 6:** (A) H9C2 cells were transfected with control siRNA (NC) or TLR4 siRNA (siTLR4) and then treated with DOX (1 μM) for 15 min. TLR2-MyD88 interactions were analyzed. Quantification is shown below. (B) H9C2 cells were transfected with control siRNA (NC) or MD2 siRNA (siMD2) and then treated with DOX (1 μM) for 15 min. TLR2-MyD88 interactions were analyzed. Quantification is shown below. (n=3; * P<0.05, ** P<0.01, *** P<0.001, vs Ctrl group; # P<0.05, ## P<0.01, ### P<0.001, vs DOX group).
